# Assessing the Safety, User Acceptability, Dissemination, and Reach of a Comprehensive Web-Based Resource on Medications for Opioid Use Disorder (MOUD Hub): Protocol for a Development and Usability Study

**DOI:** 10.2196/57065

**Published:** 2024-11-07

**Authors:** Melanie Jane Nicholls, Alexandra Almeida, Justin Castello, David Grelotti, Bianca Daugherty, Donny Gann Jr, Karen Lenyoun, Sharon Trillo-Park, Annick Borquez

**Affiliations:** 1 College of Health and Human Services San Diego State University San Diego, CA United States; 2 Programa de Computação Científica, FIOCRUZ Rio de Janeiro Brazil; 3 University Health Services University of California, Berkeley Berkeley, CA United States; 4 Department of Medicine University of California, San Diego La Jolla, CA United States; 5 Department of Occupational Therapy Colorado State University Fort Collins, CO United States; 6 Stone Soup Counseling Baltimore, MD United States; 7 School of Public Health University of California La Jolla, San Diego, CA United States

**Keywords:** opioid use disorder, medications for opioid use disorder, digital health, digital intervention, substance use, motivational interviewing, stages of change

## Abstract

**Background:**

Medications for opioid use disorder (MOUD), such as methadone and buprenorphine, are the gold standard for opioid use disorder (OUD) treatment. Owing to various barriers, MOUD access and retention are low in the United States. The internet presents a digital solution to mitigate barriers, but a comprehensive and reliable resource is lacking. We present a user-friendly, web-based resource, the *MOUD Hub,* that provides reliable information on MOUD.

**Objective:**

This study aims to assess the safety, acceptability, feasibility of dissemination, and reach of the *MOUD Hub* using focus groups and advertising on 1 key search engine and 1 social media platform.

**Methods:**

This protocol describes the development of the *MOUD Hub* and the descriptive observational feasibility study that will be undertaken. The *MOUD Hub* uses motivational interviewing principles to guide users through the stages of change. The website provides evidence-based information from national health and substance use agencies, harm reduction organizations, and peer-reviewed literature. First, pilot focus groups with 10 graduate students who have lived experience with OUD will be conducted to provide feedback on safety concerns. Then, focus groups with 20-30 potential *MOUD Hub* users (eg, people with OUD with and without MOUD experience, friends and family, and health care providers) will be conducted to assess safety, acceptability, reach, and usability. Data will be analyzed using inductive thematic analysis. The website will be advertised on Google and MOUD-specific Reddit forums to assess dissemination, reach, and user acceptability based on the total user volume, sociodemographic characteristics, pop-up survey responses, and 1-year engagement patterns. This information will be collected through Google Analytics. Potential differences between users from Google and Reddit will be assessed.

**Results:**

The *MOUD Hub* will be launched in January 2025. Data collected from 5 focus groups (approximately 30-40 participants) will be used to improve the website before launching it. There is no target sample size for the second stage of the study as it aims to assess dissemination feasibility and reach. Data will be collected for a year, analyzed every 3 months, and used to improve the website.

**Conclusions:**

The *MOUD Hub* offers an innovative theory-based approach, tailored to people with OUD and their family and friends, to increase access to and retention in MOUD treatment in the United States and provides broader harm reduction resources for those not currently in a position to receive treatment or those at risk of resuming illicit opioid use. Findings from this feasibility phase will serve to better tailor the *MOUD Hub*. After modifying the website based on our findings, we will use a randomized controlled trial to assess its efficacy in increasing MOUD access and retention, contributing to growing research on web-based interventions for OUD.

**International Registered Report Identifier (IRRID):**

PRR1-10.2196/57065

## Introduction

### Medications for Opioid Use Disorders

The opioid crisis continues to drive mortality among adults, with >100,000 drug overdose deaths in the United States in 2022 [1]. Medications for opioid use disorder (MOUD) are the gold standard for treating opioid use disorder (OUD) and reducing fatal overdose risk [2]. However, MOUD treatment is highly underused in the United States, with only 22.3% of adults with an OUD receiving MOUD in the past year [3].

Treatment with MOUD is associated with a reduction in overdose and other-cause mortality. Methadone and buprenorphine have shown similar treatment outcomes [4,5] and retention rates among people undergoing treatment across gender and racial groups [6,7] and are effective among people using fentanyl [8,9]. MOUD treatment has also been shown to lower HIV and hepatitis C virus infection risk [10-13]. Beyond reducing mortality and drug use–related health harms, MOUD treatment is associated with an increase in social functioning and an overall increase in quality of life [14]. Therefore, its uptake is warranted to ameliorate the lives of people living with OUD.

Multiple barriers to access to and retention in MOUD treatment have been identified across US settings. Knowledge gaps represent a barrier for people living with OUD, including a lack of education on available treatment options and how to access care [15]. People living with OUD report experiencing social stigma and lack of support, self-stigma, and MOUD-related stigma when accessing treatment, including poor treatment by staff and rigid treatment structures [15]. Pregnant people have expressed the increased stigma and threats they experience when accessing care that create additional barriers for them [16]. Logistic concerns such as the cost of medications, long wait times, transportation issues, and fail-first abstinence programs have been listed as barriers to accessing MOUD treatment [15]. Accessing care is especially difficult for those in rural areas due to a lack of OUD services [17].

### Web-Based Information Seeking

Given the stigmatized nature of OUD, people have been shown to seek information about detoxification, rehabilitation, and treatment through web-based resources as this offers more anonymity and confidentiality. We recently published a paper showing significant positive associations between the number of Google searches specific to help seeking for OUD (eg, “heroin detox” and “treatment for opioid use”) and indicators reflecting OUD prevalence, including the number of people receiving MOUD, the number of emergency department visits related to opioid overdose, and the number of fatal opioid overdoses across states in the United States [18]. These findings suggest that leveraging Google search algorithms could be used to effectively reach people in need of help for OUD. Similarly, web forums are a source of support, trust, and knowledge building among people living with OUD, including in the context of treatment seeking and maintenance [19]. Specifically, Reddit, a publicly available social media platform, has several subforums (known as subreddits) available to discuss and share information on opioid recovery (eg, r/OpioidRecovery, r/OpiatesRecovery, r/Methadone, and r/suboxone). As of August 13, 2024, these 4 subreddits reached >130,000 users.

Social media platforms and the internet provide a unique place for people to access information and help. People exist in different social surroundings, the first place being the home and the second place often being the workplace. Sociologist Ray Oldenburg coined the term third places, referring to the other spaces we occupy where ideas can be exchanged, community can be built, and relationships can be strengthened [20]. Examples of third places include gymnasiums, cafes, and churches. The internet has created a new web-based third place, where people can seek information, find community, and exist in ways in which they may not be able to in their first and second places. Unfortunately, public health agencies have not yet fully leveraged this resource, and much information on the internet is not based on evidence, resulting in the spread of misinformation. Misinformation has been found to spread more quickly, further, and more broadly than true and evidence-based information, as seen for vaccines during the COVID-19 pandemic [21,22]. Poor quality information on the internet has become a problem, hampering people’s ability to discern whether the information is true and providing unhelpful answers [19]. An internet resource for MOUD that offers clear, comprehensive, factual, and evidence-based information that is well tailored to the unique challenges people with OUD or their family and friends experience is necessary to increase uptake and retention.

### The MOUD Hub

We sought to create a web-based resource that provides user-friendly, reliable information to address barriers to MOUD at different steps of the care continuum, tailored to support both people living with OUD and friends and family in navigating the different stages of treatment and their respective challenges. We chose motivational interviewing (MI) as a guiding intervention. MI follows harm reduction principles to help promote progression along stages of the transtheoretical model of behavior change: precontemplation, contemplation, preparation, action, maintenance, recurrence (or relapse), and termination stages [23]. MI provides a nonjudgmental space that meets people where they are in managing their OUD and finding motivation toward behavior change.

We followed the Conceptual Framework of Access to Health by Levesque et al [24] to classify the different barriers across the MOUD care continuum, which maps onto the transtheoretical model of behavior change and consists of assessing one’s health care needs, desire for care, seeking health care, reaching care, using care, and health care consequences. The Access to Health framework comprises 5 domains characterizing health care service: approachability, acceptability, availability and accommodation, affordability, and appropriateness. These domains are connected to the 5 abilities of persons to receive health care, which include the ability to perceive, seek, reach, pay, and engage.

Therefore, we developed the *MOUD Hub*, a web-based resource to help reduce barriers to accessing and remaining on MOUD care [25]. Our goal was to create a resource that would cater to all people with OUD, including those not yet considering treatment or having stopped treatment, to facilitate linkage to services and resources across multiple needs, such as prevention services, harm reduction tools, treatment resources, and social resources. This paper describes the protocol of how this website was conceptualized and created, along with how we plan to disseminate it and assess its feasibility by characterizing its reach, use patterns, and acceptability [26].

### Study Objectives and Specific Aims

The main objective of this study is to assess the acceptability of using the website to address barriers to MOUD care and the feasibility of reaching people living with OUD and their friends and family. We first aim to gain feedback on the website’s safety, acceptability, user experience, and usefulness. After receiving this feedback and making website adjustments, we aim to assess the volume of users, their key sociodemographic characteristics, their patterns of use (content accessed, time spent with different types of content, and frequency of use), and potential differences in users’ characteristics when accessing the website from Google or Reddit. In addition, we aim to understand their views on the usefulness of the resources provided, to identify missing information and to collect web-based resources participants have found useful to further share them with the *MOUD Hub*.

## Methods

### Study Design and Setting

This descriptive observational feasibility study [26] will take place on a website called the *MOUD Hub* to investigate whether a theory-based website providing a comprehensive list of services can be helpful to people looking for information related to MOUD. The website will be advertised on Reddit opioid-related forums and through optimized Google searches with the theme of opioid use and recovery. Study outcomes will be assessed through pilot focus groups with students with lived experience of OUD who are involved in recovery groups or unions for people who use drugs, people living with OUD who have never received MOUD, people living with OUD who have experience with MOUD, family and friends of someone using opioids, and individuals who work with people living with OUD (eg, MOUD providers, counselors, and harm reduction workers); data on users will be collected in Google Analytics (Google LLC) and a pop-up survey on the website.

### Team

The website was created with the help of professionals who have expertise in substance use disorders (SUDs). The website development process was led by a substance use epidemiologist, a social worker specializing in SUD, and a digital epidemiologist specializing in SUD. This process was supported by members of a community and scientific advisory board. A clinical psychologist and harm reduction psychotherapist who works as a counselor at a university, serves as a course instructor for SUD support group facilitation, and worked as a counselor at a harm reduction organization for 14 years helped operationalize the stages of change and MI components into the website and advised on accessibility (for Spanish speakers and people with visual impairments). A social worker who works with people with SUD in hospitals and counseling practices provided insight into the harm reduction resources included in the precontemplation and contemplation stages and helped ensure that key barriers to MOUD access were addressed in the preparation and action stages. An occupational therapist with experience working with transition-aged youth with SUD informed portions of the maintenance stage of change to improve retention in MOUD treatment, including supportive apps and resources. A harm reduction advocate and mental health program coordinator with lived experience as a family member of someone with OUD provided insight into and resources on what family and friends need during this process. A psychiatrist specialized in OUD and harm reduction verified information on the medications and best practices for the recurrence and termination stages. The community and scientific advisory board members brought a diverse perspective of the population living with OUD and helped provide feedback on the creation of the website throughout the process.

### Intervention Description

#### MI and the Transtheoretical Model

Our *MOUD Hub* was built using a person-centered approach and following the principles of MI, which include partnership, acceptance, compassion, and evocation, to help individuals make decisions about their health, build a plan to problem-solve, and put them into practice. Regarding behavior change, MI is the most effective approach in changing SUD behaviors, compared to other behaviors, such as weight loss or increasing physical activity [27]. MI is a collaborative and goal-oriented style of communication that empowers people’s capacity for change [28].

The *MOUD Hub* is not only for people living with OUD but also for their families and friends. Therefore, there are specific resources for family and friends to help them understand the progression to behavior change, needs and challenges that may arise, and how to best support themselves and people living with OUD in their treatment journey. Family and friends can use the MI skills to help them encourage people living with OUD who may be seeking resources. MI seeks to meet people where they are by acknowledging ambivalence and guiding the change process [29]. It avoids using the “righting reflex” or the need to fix what is wrong with people and steer them in the right direction [29]. These concepts were integrated into the website by acknowledging the stage of change a person is in. By doing this, we offered resources to reduce harm and incorporated language that might motivate “change talk” or ignite thoughts on wanting to change.

**Figure 1:**
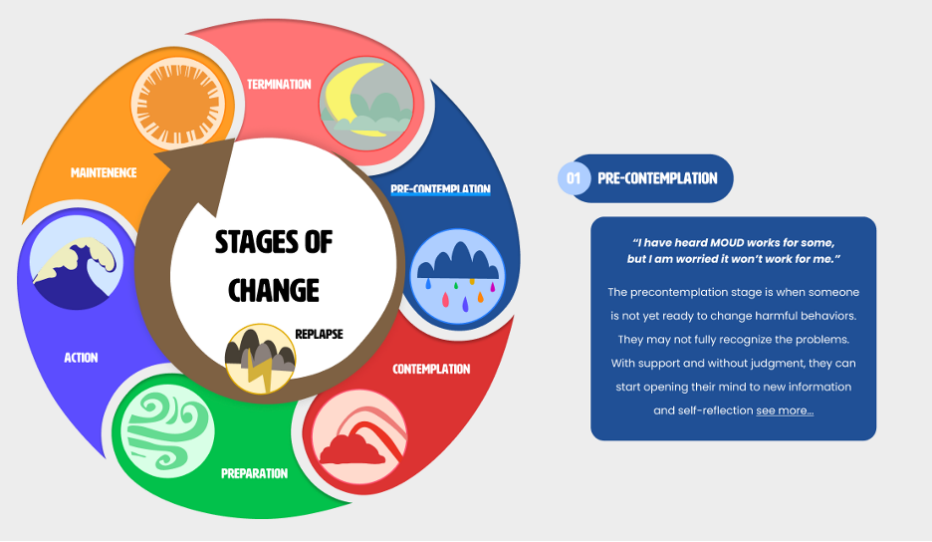
A snapshot from the MOUD Hub’s stages of change wheel. When users click on a specific stage, a pop-up appears with a brief quote and information related to that stage. Users can click “see more” and will be taken to the stage’s specific page. MOUD: medications for opioid use disorder.

MI is usually conducted in a setting with an in-person meeting between counselor and client. Using this website as a virtual third place, we integrated the concepts of MI to encourage users to elicit behavior change independently. The website follows the transtheoretical model, or the stages of change, and incorporates the skills of MI within each stage [29]. The stages of change have been used to understand that people are more likely to change their behavior when it is intentional. The stages of change are not a linear process, and each stage requires different strategies to help individuals move through them [30]. Figure 1 shows how the stages of change are introduced on the website and how information on each stage populates when clicked. When using the transtheoretical model, most research focuses on the precontemplation, contemplation, and preparation phases and has found that using stage-matched interventions helps improve recruitment, retention, and progress [31]. While several web-based resources provide information on MOUD, to our knowledge, none cover the entire progression through the MOUD treatment continuum, including precontemplation and contemplation stages preceding engagement. In addition, the stages of maintenance and concerns such as substance use recurrence are rarely attended to in these interventions. We identified issues and challenges specific to each stage of change and resources that would benefit users in each stage. For more information on how the stages will be used and example pages of the website for each stage, refer to Multimedia Appendix 1.

As in an MI session with a counselor, the *MOUD Hub* does not indicate to the user which stage of change they might be at and instead relies on their self-identification based on the stages’ descriptions and associated quotes provided on the home page. The reasoning behind this is 2-fold. First, we wanted to stay aligned with the principles of MI, including the horizontal relationship between the client and the counselor and the respect for the client’s capacity to identify the right tools for themselves (rather than a top-down educational approach in which the counselor informs the client of their condition and the steps they need to follow to resolve it). Second, some users might be friends or family of people living with OUD, and it might be harder for them to accurately complete an assessment, leading to inaccurate guidance. In both cases, this open approach might lead users to explore several stages before focusing on one stage, which could increase their understanding of the process of change.

#### Resources Provided and the Conceptual Framework of Access to Health

The *MOUD Hub* consists of a repository of evidence-based resources and links to web-based material to address barriers to MOUD treatment engagement and retention. To identify the challenges for which resources were needed, we conducted literature searches on MOUD barriers and facilitators and complemented these findings using insights from Reddit (A Almeida, personal communication, 2024). Specifically, natural language processing methods were applied to Reddit textual data to understand the main struggles faced by people who use MOUD throughout their treatment (A Almeida, personal communication, 2024). We used the results of the topic models that unveiled the main issues discussed in the r/Methadone and r/suboxone subreddits to guide sections of the *MOUD Hub* and help identify the resources needed. For example, the topic “Back to suboxone after relapse” guided the incorporation of links and materials in the *MOUD Hub*, elucidating the safest and best practices to resume suboxone treatment. The purpose of using this Reddit analysis to inform the website was to better cater to the needs of people seeking help on the web to address OUD, as their demographics and circumstances may differ from those not seeking help on the web.

Resources were obtained from verified sources that specialize in substance use, such as the Substance Abuse and Mental Health Services Administration, Centers for Disease Control and Prevention, and websites connected to the National Institute on Drug Abuse, as well as harm reduction organization websites, including the National Harm Reduction Coalition. Peer-reviewed papers were used as references for information to ensure that the content was based on evidence and supported by research. References are cited for every resource provided so that users can access information at its source.

**Figure 2:**
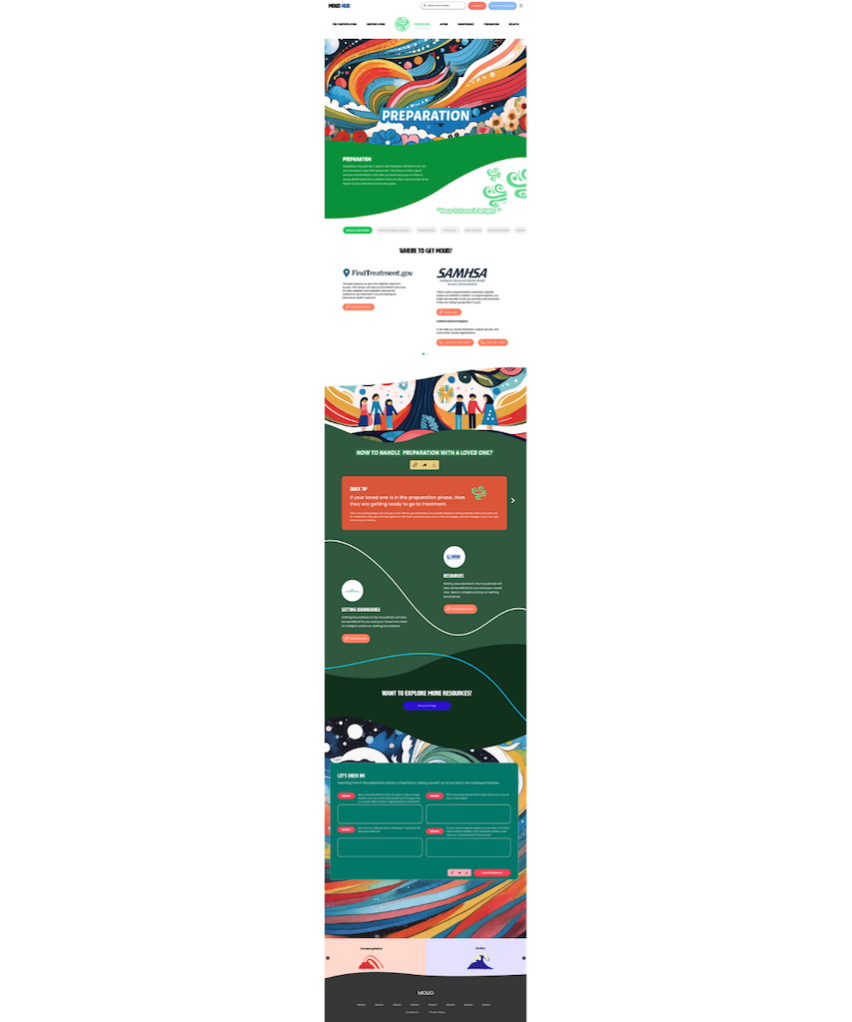
A snapshot of the preparation stage home page of the MOUD Hub. Each stage has a home page with a description of the stage and a tailored affirmation at the top, followed by tabs for different themes relevant to the stage. For the preparation stage, the themes are where to obtain medications for opioid use disorder (MOUD), recovery support groups, transportation, pharmacy, cost of MOUD, storing medication, treatment during pregnancy, preparing for stigma, who can I call in a crisis, friends and family, and check in. SAMHSA: Substance Abuse and Mental Health Services Administration.

**Figure 3:**
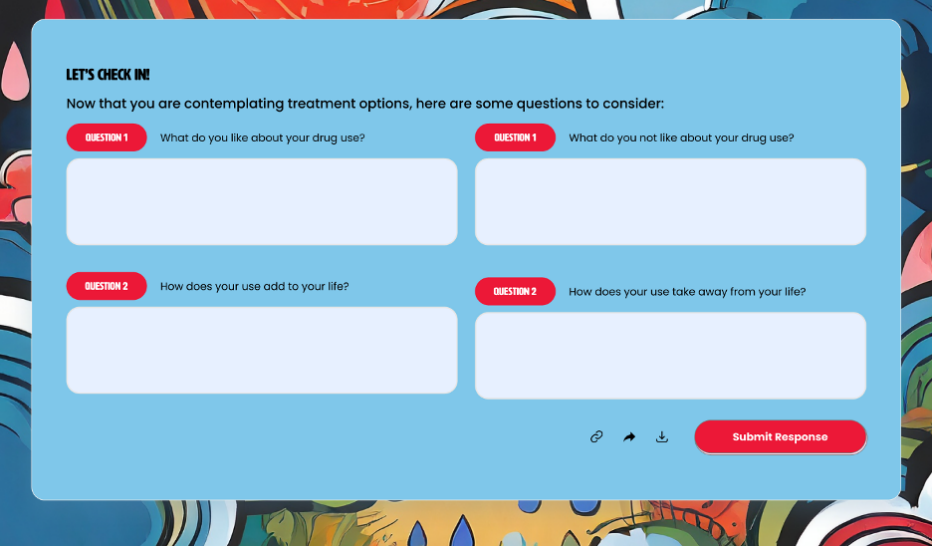
An example of a check in box for the precontemplation stage. Each stage has a tailored check in with questions guided by the principles of motivational interviewing. Users will be able to answer the questions and download them to their personal devices.

Resources were selected to address the challenges that usually present at each stage of the transtheoretical model and were classified using the Access to Health framework, assessing resources’ fit into the 5 dimensions, including approachability, acceptability, availability and accommodation, affordability, and appropriateness [24]. The corresponding abilities of persons were considered when assessing barriers and resources that would be helpful. Table 1 shows an overview of the stages, the barriers that people living with OUD face in each stage, and the resources shared to remedy those barriers. Each barrier and resource are connected to the domains of the Access to Health framework and the MI open-ended questions used to elicit behavior change. Figure 2 is an example of the main preparation stage page and its resources. Figure 3 is an example of a stage-specific check-in box that uses MI questions. Refer to Multimedia Appendix 2 for the complete series of web pages for all stages at website inception.

**Table 1:** Stages of change and related barriers or issues as found in the literature, the resources provided to address these barriers, how the resource relates to the access to care framework, and relevant motivational interviewing (MI) questions included in the check-in box.

Stage of change and barrier or issue		Resources provided	Access to Health framework by Levesque et al [24]
**Precontemplation stage^a^**			
	Limited understanding of OUD^b^ [15]	What is OUD? What do opioids do to the brain?	Ability to perceive
	Lack of trust in MOUD^c^ [15]	MOUD myths dispelled	Acceptability Ability to perceive
	Lack of knowledge or understanding of harm reduction [32]	Harm reduction resources (safe injection practices, syringe services, overdose prevention, wound care, drug checking, HIV and HCV^d^ testing)	Approachability Ability to perceive
	Lack of readiness to start MOUD [32]	Harm reduction resources to help in using drugs more safely	Acceptability
**Contemplation stage^e^**			
	Lack of knowledge of MOUD options [15,32]	Resources and information on the different types of MOUD (methadone, buprenorphine, and naltrexone)	Approachability Acceptability Appropriateness Ability to perceive Ability to seek
	Strong preference for detox versus maintenance treatment [32,33]	Web-based Information on detox (including risks) and advantages of maintenance	Ability to perceive
**Preparation stage^f^**			
	Lack of knowledge on where to access treatment [15]	Treatment locator Helplines	Approachability Availability and accommodation Ability to seek
	Logistic issues [15,32]	Transportation help Storing medication Insurance coverage	Acceptability Affordability Appropriateness Ability to reach Ability to pay
	Stigma around OUD and MOUD [15,32]	Preparing for stigma Responding to stigma Recovery support groups	Acceptability Ability to engage
	Concerns about treatment during pregnancy [16,32]	Information about benefits of treatment during pregnancy and relevant state policies	Approachability Ability to perceive
**Action stage^g^**			
	Coping skills during early treatment [32]	Information on different coping skills	Approachability Ability to perceive Ability to engage
	Dealing with side effects of medications [34]	Resources on strategies to address side effects	Availability and accommodation Ability to perceive Ability to engage
	Lack of strategies to prevent recurrence [32]	Creating a recurrence prevention plan	Ability to engage
**Maintenance stage^h^**			
	Lack of social support during treatment [15,32]	Apps for support and MOUD support groups	Appropriateness Ability to engage
	Trauma or mental health concerns [32]	Mental health support and where to find mental health treatment	Availability and accommodation Ability to seek
	Boredom when no longer using illicit opioids [32]	Finding new sources of pleasure Create a recurrence prevention plan	Approachability Ability to engage
**Termination stage (ending MOUD)^i^**			
	Strong desire to terminate treatment	Referring to a physician for support and to prevent overdose Reiteration of the importance of long-term treatment	Approachability Acceptability Appropriateness Ability to perceive Ability to engage
**Recurrence (or relapse) stage^j^**			
	Stigma or shame around recurrence. The belief that recurrence is a “failure” [35]	Research on recurrence as a natural occurrence of OUD and opportunity for readjustment	Approachability Ability to perceive Ability to engage
	Belief that recurrence is long-term	How to return to treatment	Approachability Acceptability Appropriateness
	Lack of strategies to prevent recurrence [32]	Create a recurrence prevention plan	Ability to seek Ability to engage
	High risk of overdose during recurrence	Revisit harm reduction resources	Approachability Ability to perceive

^a^Precontemplation stage MI check-in questions: readiness rulers [36]—How important is it for you to change your opioid use? What made you choose that number and not a lower one? What would help you pick a higher number? How confident are you in making this change? What would help you be more confident?

^b^OUD: opioid use disorder.

^c^MOUD: medications for opioid use disorder.

^d^HCV: hepatitis C virus.

^e^Contemplation stage MI check-in questions: How does your current opioid use affect your life, relationships, career, and future goals? How would treatment help you resolve some of those and help you achieve your goals?

^f^Preparation stage MI check-in questions: What are the ways to address some of the challenges you are worried you may face? What are the steps you may need to take to receive treatment?

^g^Action stage MI check-in questions: What has been working well for you? Are there any places that you need to strengthen to help your progress?

^h^Maintenance stage MI check-in questions: What challenges have you been encountering? What are practical ways to address them? Can you name 3 things that have improved since starting treatment?

^i^Termination stage MI check-in questions: readiness rulers [36]—How confident are you in making this change?

^j^Recurrence (or relapse) stage MI check-in questions: How has this relapse gotten me closer or farther away from my treatment goals? What do you think you need to help you in your next steps?

Apps deemed useful, such as the Calm App, were added as resources. Although we recognize that some apps may not be tested through randomized controlled trials, we chose those that have been based on helpful practices, such as mindfulness and harm reduction [37]. The *MOUD Hub* informs users that these apps may not be evidence based but that they might find some of them useful when trying to use coping skills for their treatment.

The website explicitly includes disclaimers regarding the fact that it does not offer medical advice; the language used, which in some instances includes terms from other resources that may be considered stigmatizing and for which we have used quotation marks; and noting that the resources are compiled rather than created by us (Multimedia Appendix 2).

### Accessibility

The website has tabs to organize how users access the resources. For example, there is a “Languages” tab showing users how to set up and enable translations in their web browser if English is not their preferred language, as well as an “Audio” option that informs users on how to have the website read to them to reduce access barriers among those with vision or literacy concerns. The website was developed to be accessed from a computer and a mobile phone to increase accessibility.

### Dissemination

The dissemination of the *MOUD Hub* will follow web-based marketing strategies, which harness interest in a topic, as expressed by internet searches or membership to specific social media networks, to promote the resource and increase its uptake. We will use internet searches on Google and leverage Reddit subreddits or discussions focused on opioid use recovery, treatment, and overdose prevention.

For Google, we will advertise this website in response to specific searches using sets of words described by Patton et al [18]. For example, when someone googles “opioid+treatment,” they will see our advertisement. For Reddit, the website will be posted monthly in the subreddits of r/Methadone, r/suboxone, r/OpiatesRecovery, and r/Naloxone. We will advertise the *MOUD Hub* monthly and tailor the post to incorporate the month’s most discussed topic. The monthly frequency of the posts is driven by the subreddits’ constraints on the maximum number of research-related posts.

### Recruitment, Sample, and Participants

To evaluate the safety and preliminary acceptability before advertising the website, we will first carry out pilot focus groups with 10 graduate students with lived experience of OUD, personally or through family and friends, and who are participating in recovery groups or unions for people who use drugs. In these focus groups, we will pay particular attention to safety and make changes to the MOUD Hub accordingly. Participants will be recruited by contacting UC San Diego and San Diego State University student recovery groups.

After the pilot focus groups have been conducted and relevant changes have been made, 4 additional focus groups will be conducted with people living with OUD who have never received MOUD; people living with OUD who have experience with MOUD; family and friends of someone using opioids (including people living with OUD); and individuals who work with people living with OUD, including MOUD providers, counselors, and harm reduction workers. Each focus group will aim to have 4 to 8 participants. Participants will be recruited from MOUD treatment centers, support groups for family or friends of people living with OUD, and harm reduction organizations in San Diego through email to ask for permission to share study fliers. The purpose of these focus groups will be to gain more in-depth knowledge on the safety and acceptability of the hub, user experience, and thoughts on its usefulness in improving MOUD access and retention.

For all focus groups, participants will have the option of engaging in a focus group either through Zoom (Zoom Video Communications) or in person. Inclusion criteria for participants include being aged ≥18 years and having at least one of the aforementioned experiences. Exclusion criteria include experiencing severe mental health issues and being unable to provide informed consent.

There are no eligibility criteria for the study when assessing the website’s reach due to it being available for public access. We will be openly advertising it to people who make relevant Google searches or who are members of opioid-related subreddits. Similarly, there is no randomization, as all users can access the same website’s content. We will be able to identify whether participants came from Reddit or Google and treat them as 2 different samples. This will enable us to investigate differences in user volume and characteristics when accessing the website through Reddit or Google and to evaluate the most appropriate platform for dissemination for future-related interventions.

There is potential for bias, including misclassification, self-selection, and response bias. It will not be possible to know whether users are experiencing OUD or searching for information for a loved one, limiting our understanding of the representativity of the sample by OUD experience and leading to potential misclassification bias. However, we will be able to know whether users clicked on resources specific to friends or family and whether they responded to the “check-in” questions directed at people living with OUD. In addition, the pop-up survey asks whether users are accessing the website for themselves or a friend or family member. This will provide us with an additional measure for the proportion of users who are seeking help for themselves versus for a friend or family member.

Effectively, all people accessing the website will self-select based on need or curiosity, and we will miss information from people living with OUD who are not seeking help for OUD or are not compelled to access the *MOUD Hub* when exposed to it. The demographics data will allow us to assess to what extent our sample is representative of the general population and the population experiencing OUD and fatal overdoses in the United States in terms of geography, age, and gender. We will also be able to assess whether users are accessing the website from Google, Reddit, or other sources, potentially representing different communities. While response bias is possible in the context of our pop-up survey, participation will be voluntary and anonymous, and therefore, this will be minimized. However, there will undoubtedly be self-selection bias among the group answering the survey, likely representing those most engaged in improving access to reliable MOUD information*.*

### Outcomes and Measures

To evaluate the safety and acceptability of the *MOUD Hub*, we will conduct pilot focus groups with 10 graduate students belonging to recovery groups or unions for people who use drugs. We will lead 2 focus groups to explore safety-related issues, along with gaining feedback on the acceptability, content, and any additional information that should be provided on the website. Safety concerns will focus on, but will not be limited to, information that may potentially be triggering or misleading and stigmatizing language.

A total of 4 additional focus groups will be conducted with other groups to gain more in-depth knowledge on the acceptability of the hub, user experience, and thoughts on its usefulness, following improvements from the pilot focus group. The topic guide in Multimedia Appendix 3 will be used.

To evaluate the reach and use patterns of the website, we will use Google Analytics to collect data on the number of users, their characteristics, and their interactions with the website [38,39]. The website will be launched in January 2025, and we will collect data for evaluation for a year and look at the data in 3-month increments. With this iterative approach, we can adjust the website in real time.

Our protocol’s outcomes and measures are shown in Table 2. We will first assess the dissemination reach of the website. The reach of an intervention is defined as “the absolute number, proportion, and representativeness of individuals who are willing to participate in a given initiative, intervention, or program, and reasons why or why not” [40].

**Table 2:** Outline of the quantitative and qualitative measures that will inform the outcomes. Each measure is labeled with the information’s source.

Reach		Acceptability
**Quantitative measures**		
	Volume of users (GA^a^)	Proportion of users who return to the website (GA)
	Average time spent on the website (GA)	Acceptability of Intervention Measure questions (PS^b^; Figure 4)
	The average number of resources the users clicked on in different stages (GA)	—^c^
	Total number of times a resource was clicked within each stage (GA)	—
	User demographics (age, gender, location, language, and accessing the website for themselves or for a friend or family member; GA and PS)	—
**Qualitative measures**		
	Focus group interview guide assessing reach: Do you think people would use the website? Who do you think would use the website? (FG^d^)	Open-ended questions about acceptability; for example, “What did you find most helpful?” and “What were you expecting to find that was missing?” (PS; Figure 4)
	Focus group assessing who it would be useful in reaching: Who do you think would use this website the most? (People living with OUD, family, friends, SUD^e^ professionals?)	Focus group interview guide assessing safety, acceptability, usability, and further feedback on usefulness. Example questions are “What are your overall impressions or thoughts on the website?” and “Is there something on the website you particularly liked/disliked?” (FG)

^a^GA: Google Analytics.

^b^PS: pop-up survey.

^c^No additional information.

^d^FG: focus group.

^e^SUD: substance use disorder.

**Figure 4:**
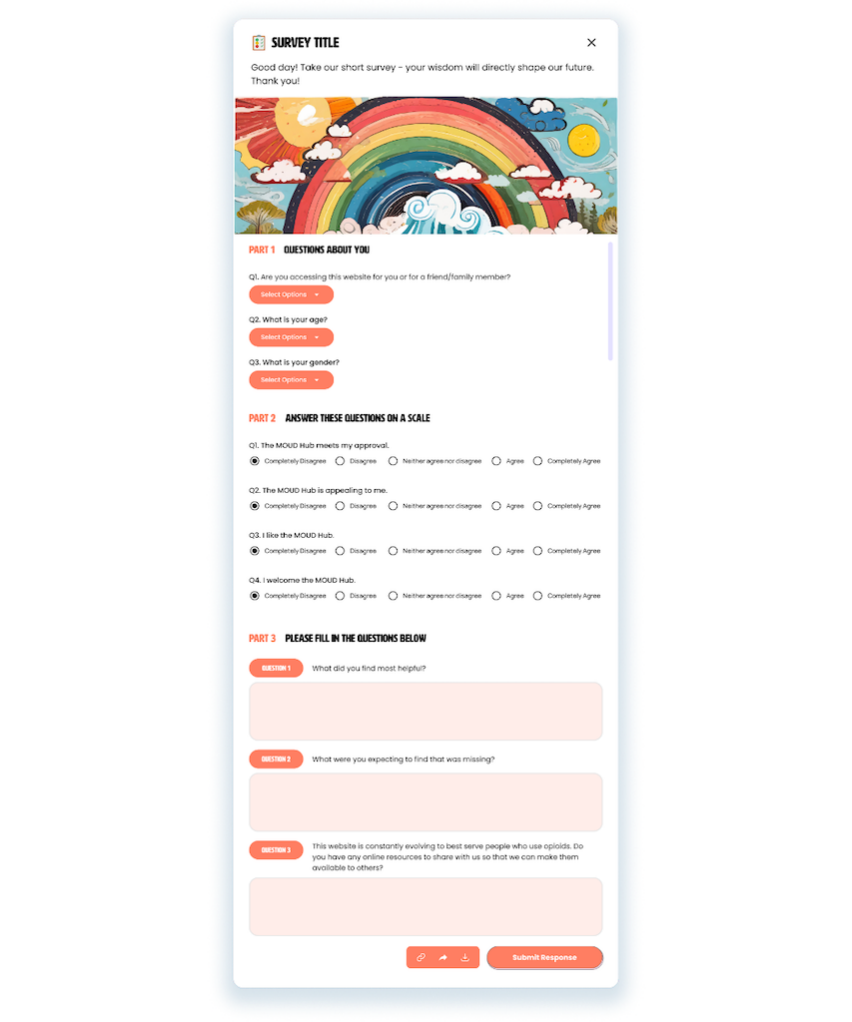
A pop-up survey that will be presented to users before they leave the website to collect demographic information and initial acceptability of the website. MOUD: medications for opioid use disorder.

We will measure the volume of users who access the website and user demographics to characterize who we are reaching. We will measure the average time spent on the website, the average number of resources the users clicked on, and the total number of times each resource was clicked on. This will help determine whether users genuinely engage with the website and identify which stages and specific resources are most relevant to them. Google Analytics provides data on how many users visit the website, when they visited, how long they stayed on the website and on specific pages, and what resources they clicked on, along with users’ age, gender, location, and language preferences. Information on demographics, such as age and gender, and whether the users are visiting the website for themselves or for a friend or family member will also be asked in a pop-up survey that will appear when people leave the website (Figure 4).

To further assess acceptability, we will measure the proportion of users who return to the website along with using the Acceptability of Intervention Measure [41]. The pop-up survey will ask closed- and open-ended questions to assess acceptability and identify additional relevant resources. The closed-ended questions will use the 4 questions from the Acceptability of Intervention Measure [41]. The open-ended questions will ask about ways in which they found resources helpful, whether there are other topics or questions that they expected to obtain information on but did not find anything relevant, and whether there are any other resources they would like to share with the broader community. Google Analytics tracks the number (and proportion) of people who are returning users, which will be used as another measurement of acceptability.

Finally, we will compare users who come from Reddit versus those who come from Google by looking at the volume of users from each source and their characteristics. The aforementioned outcomes will be assessed to compare the effectiveness of social media versus search engines in disseminating resources such as the *MOUD Hub.*

For the website’s reach, we hypothesize that (1) users will most frequently click on links related to the precontemplation or contemplation phases of MOUD use, as opposed to links related to the preparation, action, and maintenance phases; (2) a substantial proportion of users (>25%) will be family members or friends of people who use opioids; (3) the volume of users referred through Google searches will be larger than that of users referred through Reddit; and (4) users referred through Reddit will spend more time on the website and click on a higher number of links than users referred through Google searches.

For acceptability, we hypothesize the following: (1) most users will find the resource useful and ratings will be significantly higher among acquaintances of people who use opioids than among people who use opioids themselves; (2) users with OUD will find the resource less useful; and (3) a large proportion of resources shared by users will lead to non–evidence-based information, including rehabilitation centers based on mandatory abstinence.

### Data Collection and Management

For all focus groups, each potential participant will first do a Zoom screening session where the consent form will be read to them; basic sociodemographic information (ie, age, gender, and race or ethnicity) will be collected; and they will be asked about their availability for the focus group from a list of potential dates and times. The consent form outlines the study’s components, the risks and benefits of enrollment, the length of time for the focus group process, the duration of the study, and contacts for the study and university should the participant have a question. As part of the consent form, participants will be informed that their “physical” identity will not remain anonymous with the other participants as they will see each other on Zoom or in person. Verbal informed consent will be obtained and recorded from eligible participants before starting the focus group.

Focus groups will be conducted either on Zoom or in person. For Zoom focus groups, each person will be sent an invitation to a password-protected Zoom room, and each will be added to the Zoom room one by one by the researchers, ensuring no names are shown on their profile. In-person focus groups will be hosted at UC San Diego, either in La Jolla or Hillcrest, depending on the participants’ preference, in a private room where others cannot listen in.

For all focus groups, a topic guide has been created that covers topics of the websites’ safety, acceptability, user experience, reach, usefulness, safety, and additional resources or suggestions that participants have (Multimedia Appendix 3). Focus groups will last for 60 to 90 minutes and will be audio recorded on a digital Dictaphone (no images or video will be recorded). Participants will be compensated with a US $20 debit card. Data from the focus groups will be kept on a password-protected computer with restricted access by the research team and transcribed.

Google Analytics will provide information on users’ general demographics, site of origin (Reddit or Google), and the time spent on each web page. All data analyzed to investigate the *MOUD Hub* dissemination potential are from Google Analytics and website traffic. Google Analytics will provide details about the users, such as whether they are new or returning; their session on the website; what website pages they visit; page views; and bounce rates, which is when a user visits a website and leaves without interacting with any other pages [42]. Data will be stored on a password-protected computer with restricted access to the research team. Access to the Google Analytics data is username- and password-protected.

### Statistical and Qualitative Analyses

Focus group transcripts will be analyzed using inductive thematic analysis. This inductive approach is useful when using direct questions to describe a particular experience and find common themes within data [43]. Two researchers will go through multiple coding iterations, with the first consisting of coding the data without preconceived categories. After the first round of coding, the researchers will decide on what codes to use and apply finalized codes using Atlas.ti (Lumivero) software [44]. Consistency will be achieved through regular team meetings, coding disagreements will be resolved through team deliberation, and code definitions will be enhanced as needed [45,46]. An in-depth review of coded data will lead to the identification of themes related to the safety, acceptability, and potential reach of the MOUD Hub and its hypothesized usefulness in improving access to and retention in MOUD treatment. We will then conduct member checking with selected participants to reduce the risk of data misinterpretation [47,48]. We will keep a log of suggestions and reasons regarding why they were or were not implemented.

Open-ended responses to the pop-up survey in the MOUD Hub will be analyzed on Atlas.ti software by the same 2 researchers using deductive-inductive thematic analysis based on the codebook developed for the analysis of the focus groups’ data but allowing for new codes to emerge [49]. The same process will be followed to reach a consensus on the coding and to identify themes to answer our research questions.

Descriptive statistics will be conducted to assess the population that has accessed the website, their website of origin (Reddit or Google), how long participants were on the website, and how many pages they visited. Descriptive statistics will include the percentages, means, and SDs of age, gender, and geographic location of users. Descriptive statistics on how long participants were on the website and how many pages they visited will be measured by the total number of times the web page was visited, how many pages were visited, and the proportion of returning users. Descriptive statistics on the mean and SD of responses to the Acceptability of Intervention Measure will be assessed, with a mean between 4 and 5, corresponding to the “agree” and “completely agree” options in the scale, interpreted as users finding the website acceptable. We will produce CIs for each outcome.

Bivariate statistics, such as 1-tailed *t* tests, will be used to examine the difference in outcomes between users who come from Reddit and those who come from Google. Similarly, we will investigate differences in sociodemographic characteristics and use patterns of the website by conducting bivariate *t* tests. We will apply statistical tests to identify significant differences in the number of times each stage was accessed (vs all other stages) and in the number of times each specific resource was clicked on (vs other resources within that stage).

### Ethical Considerations

Following implementation science guidelines, we will first assess whether this resource is acceptable and can be disseminated effectively and equitably before testing whether it can help with behavior change in a randomized controlled trial. Overall, there are minimal safety considerations at this time due to all resources provided coming from trusted sources and evidence-based studies that are accessible through web-based resources already. However, safety considerations will be thoroughly assessed during focus groups.

There is no disclosure from people accessing the website. Participants will not be identifiable as we will not collect personal identifiable information or geographical data that could exactly locate them. The resources and tools on the website are labeled as nonmedical advice, and participants are always informed to consult a physician. The study has been exempted by the University of California San Diego Institutional Review Board (protocol# 809353) due to the information being focused on the assessment of an educational resource as opposed to participants’ personal experiences, and no personal identifying information will be collected. We will, nonetheless, take participants through an informed consent process for rigor and transparency.

## Results

This research was funded by the National Institute of Health Avenir Award (NIH DP2 DA049295) in July 2019. The *MOUD Hub* will be launched in January 2025. We do not have a target sample size as this is a descriptive, observational, feasibility study aiming to gain information on the dissemination and reach of the website. Data will be collected for 1 year (from January 2025 to January 2026) and assessed in 3-month increments. Results based on the *Outcomes and Measures* section will be used to modify the website accordingly at each 3-month period. Results will show the evolution of the website and access patterns over time and will be published after the full year of assessment.

## Discussion

### Expected Findings

We have described our planned protocol for assessing the safety, acceptability, and reach of the *MOUD* *Hub* website, which will be used as a resource guide for people seeking information on MOUD. This website is guided by harm reduction and MI principles, the stages of change, and Access to Health frameworks. We believe that it will be a helpful guide for those who experience barriers to accessing relevant information and care.

Our website includes information on evidence-based practices and interventions for users to consult and interact with. We believe that web-based platforms are a virtual third place for people to occupy and can provide knowledge and community building for those who may feel isolated and stigmatized. We expect to characterize users’ interactions with the information provided and to gather their opinions on the usefulness of the resources for MOUD treatment.

Once these suggestions are incorporated into the website, we aim to conduct a randomized controlled trial to test the efficacy of the website in improving the uptake of and retention in MOUD. There are a few potential designs to help assess the intervention’s efficacy. The first option would be to test the efficacy of the *MOUD Hub* by assessing differences in MOUD uptake and retention among people accessing the website. This could be done by recruiting participants on Reddit and Google based on their search for help, addressing their OUD, randomizing them to receive the *MOUD Hub* (ie, the intervention) or the Substance Abuse and Mental Health Services Administration website (ie, the control), and following up over time by asking them to fill in surveys regularly and obtaining access to their medical records to assess their treatment progress.

A more rigorous design would be to physically recruit participants and follow their treatment progress regularly. This type of analysis would allow us to compare efficacy among those stably housed and unstably housed to further assess whether a website is a helpful resource for those who might need to access the internet in public spaces versus the comfort of their own home.

Another possibility to assess the efficacy of the *MOUD Hub* could be to work with emergency departments. Participants could be those who are screened for an OUD, came in due to a recent overdose, or came in due to complications from their use. Participants would be randomly assigned to the *MOUD Hub* as a resource to learn more about MOUD in addition to the treatment-as-usual approach (eg, referral to OUD treatment) versus a treatment-as-usual approach only. Participants would be followed up to assess their uptake of (and retention in) MOUD.

Across study designs, qualitative interviews would be conducted to explore how the participants used the website, whether it impacted their decision to seek out MOUD, and whether it played a role in the participants taking and staying on the medication. This could inform future health research on whether a health information website that incorporates MI and the Access to Health framework can be more beneficial than those that only provide information.

A long-term goal will be to incorporate a discussion forum on the website for people to anonymously engage with others sharing similar questions and concerns, where they can share other resources, discuss their MOUD treatment, and offer support. There is potential for this website to become an “app” that can provide more personalized information and support for users, such as hotlines, trackers to help users see their progress, notifications to help keep users motivated, and space for reflections on their treatment.

### Limitations

One key limitation of this study is recognizing that not everyone has access to the internet, and thereby, some people will be excluded from using the *MOUD Hub*. Those most vulnerable often lack regular access to the internet. Therefore, if the *MOUD Hub* was successful in supporting access to and retention in MOUD treatment, it could exacerbate existing disparities in MOUD coverage by socioeconomic and housing status. However, among those who are unhoused or not working, the internet can be more accessible compared to physical services, with recent studies finding that >50% of adults experiencing homelessness have phones with internet access [50].

Web-based platforms offer a lifeline or support to people who may not be able to access health care regularly. The internet is a powerful tool in helping patients collaborate with their physicians by being more active in decision-making, preparing for their appointments, and facilitating communication [22].

Another limitation at this stage is that the website is not interactive, thus losing parts of the spirit of MI that can only be done through a person-to-person conversation. However, we are using elements of MI that might help the website users take the first step to receive the support needed. For example, we provide education and MI check-in questions tailored for each stage, including decisional balance questions and readiness and confidence rulers. Users will be encouraged to write their reflections on these questions in the space provided, and the website allows them to download their responses for them to keep. It is well established that information is not enough to elicit behavior change and that other issues take over when it comes to accessing SUD treatment (and other health interventions). However, we are providing concrete resources to address mistrust and logistical issues and ensuring that people with OUD, as well as their friends and family, are well equipped to understand how MOUD works and how to access and remain on treatment as an important first step.

Another limitation is that the *MOUD Hub* is not personalized at this stage, meaning that users cannot create a profile or track their progress. The website will not be able to follow a user’s progress through the stages of change or keep a record of the resources that are useful for a specific person at a specific point in time. However, due to the website being in its first stages of development, this may be explored later and added to test whether personalization benefits users.

In addition, due to the website having a US-wide scope, the resources are general rather than specific to each setting, and it is up to the user to take steps to locate resources in their area if they are not included in the national MOUD, syringe service programs, or naloxone locators, for example. Finally, using Google Analytics poses a limitation as it estimates users’ demographics through algorithms as opposed to self-report or routine databases.

### Conclusions

This is the first website to provide comprehensive, reliable, and evidence-based sources on MOUD, considering all stages of change and catering to both people who use opioids and their friends and family. We trust this resource will increase awareness of MOUD, improve knowledge, dispel myths about its treatment effects, and support patients and families in accessing treatment. Future directions will focus on testing the efficacy of the *MOUD Hub* in increasing the uptake of and retention in MOUD.

## Appendix

Multimedia Appendix 1 Additional information on the stages of change.

Multimedia Appendix 2 High-fidelity snapshots of the web pages.

Multimedia Appendix 3 Interview guide for focus groups to assess the website.

## Abbreviations

MI: motivational interviewing
MOUD: medications for opioid use disorder
OUD: opioid use disorder
SUD: substance use disorder

## Data Availability

The datasets generated during and analyzed during this study will be available from the corresponding author upon reasonable request.
