# Supplementary material for: Assessing the Safety, User Acceptability, Dissemination, and Reach of a Comprehensive Web-Based Resource on Medications for Opioid Use Disorder (MOUD Hub): Protocol for a Development and Usability Study
Source: JMIR Res Protoc. 2024 Nov 7;13:e57065. doi: 10.2196/57065 (PMC11582488; doi:10.2196/57065)
Supplement: Multimedia Appendix 3 [file resprot_v13i1e57065_app3.docx]

Interview Guide for Focus Groups

Introduction: Thank you for taking the time to meet with us today. We have an MOUD Hub website that some of you may have already gotten to use, while some of you will be seeing it for the first time. For the purpose of today, we want to get your feedback on your user experience with the website, how useful you find it, and any additional resources or topics that we should include. So let’s get started.

Please be honest, if you do not find it useful or interesting, etc, please tell us as we want this to be useful for people.

Introduction:

1. If this isn’t your first time seeing this website, how did you first come across it? [When did you first come across the website, how much time have you spent looking at it, what do you most remember about it, and please be honest with your answers]
   1. Google, reddit, etc?
2. Can you tell us everything you remember about it? [Getting a sense of whether they went through it, caught the stages of change, if they mainly speak about the design and colors, capturing the most striking features]
3. Before we start getting into the details, we wanted to get a sense of whether you identified any safety concerns as you browsed through it. [anything that was triggering, stigmatizing, or offensive]

Before jumping into other questions, orient them to the website, if they have not seen the website. Go through how it is formatted, how the stages of change are incorporated, and if they recognized it. We will first go over the design of the website and then ask about the content.

General impressions, after going through some of the website with each group and seeing their thoughts on parts of the website.

Acceptability/User Experience Questions: First we will talk about the overall website user experience.

1. What do you think of the name “MOUD Hub?”
2. What are your overall impressions or thoughts on the website?
   1. What is something that struck you the most on this website?
   2. Is there something on the website you really liked?
   3. Is there something on the website you disliked or felt was unnecessary/unhelpful?
3. What do you think of the design?
   1. Does it seem acceptable for all ages and people?
   2. What do you like/don’t like about it?
4. What do you think of the color scheme? [Are the colors too bright, too much, or do they add a vibrancy and positivity to the website?]
5. What do you think of the weather theme? Did you notice it?
6. Do you think there is too much text? What do you think about the amount of text?
   1. Is the amount of information enough, too much?
   2. What do you think of the size of the font? Is it easily readable?
7. What do you think about the affirmations? Did you notice them?
   1. Are there any affirmations that you think would be better suited?
8. Would an interactive AI piece that could ask questions or direct users to different parts of the website be helpful?
9. How is the overall user experience?
   1. How did you find the experience navigating the website?
   2. What is your take on the overall organization of the website? [Organized by the stage of change and topics, does it make it difficult to access information, does it make it less likely that people will see relevant information]
   3. Are there any areas that do not work correctly that you have found? Such as broken links, or pages do not work correctly?
10. What would you rate the usability of the website on a scale of 1 not being usable and 10 being easy to use? Why not a higher score?
11. Are there any features that could be added? What about features that could be taken out?
12. How could we make it more engaging?
    1. Is the language acceptable? What are your thoughts on the language used? [Probe: do you find any of it stigmatizing, disrespectful, etc]
    2. What are some words, topics, or themes we should not use due to them being stigmatizing?
    3. Is it using suitable language for people who might use it? [is it easy to understand, accessible language]
13. When looking at the information and resources that are shared, are there any things that might get pushback/negative reactions from people visiting the website?

Reach Questions:

1. Do you think people would use the website? Who do you think would use the website?
   1. Who do you think would use this website the most? [PWOUD, family, friends, SUD professionals?]
   2. In terms of severity of opioid use who in that spectrum are the ones you could see engaging with this website? Are we completely losing people who have a severe opioid use disorder

Usefulness Questions: Now we will move on to the content of the website.

1. Would this website be useful to you? How so? [Would it actually be helpful for anyone? What about for family or friends?]
2. Who do you think this website could be useful for, if you can think of anyone?
   1. Things that would make people lose trust in the website?
   2. Are there any topics, language use, or anything else that might cause someone to lose interest in the website?
   3. What about family and friends?
3. How do you feel about using the stages of change to guide through the website?
   1. Is it useful?
   2. Would it be helpful to have an assessment for users to see what stage they might be in?
4. What are your thoughts on the use of the stages of change? (Does the flow of it make sense, would you follow it?)
   1. Are they helpful? In what ways? How are they not helpful?
   2. When looking at the stages of change, do you think they would help you/a loved one understand where they are the change process?
   3. Are we addressing the right challenges in each section?
5. Do you see yourself coming back to the website and utilizing the different stages of change and resources?
   1. Do you think this can help someone move through the steps?
6. What are your thoughts on the check-ins? How do you think people might engage with the check-ins?
   1. Do we need to make it more interactive? If so, how could we make it more interactive?
7. How do you think this website adds to the information already available on the internet about MOUD? [if at all]
   1. How do you think this is different from other websites that share similar information? (SAMHSA, local recovery centers)

Any additional barriers or resources: Lastly, we would like to get your thoughts on whether we are missing anything, whether it be barriers that you have come across or resources that have been helpful for you that were not listed here.

1. Is there a resource or concept that we are missing?
2. What other barriers have you come across when it comes to MOUD that maybe aren’t listed here?
3. What information have we shown that you do not agree with or that you feel isn’t completely useful or trustworthy?
4. Are there any additional resources that have helped you with accessing MOUD?
5. Any resources that could be helpful in overall recovery?
   1. Podcasts, books, support groups
6. If you could design a website for people who use opioids or their family, how would you do it, what would you put in it?
7. How do you feel about providing books, podcasts, and apps that aren’t evidence-based [or might be more abstinence based]?

Safety Questions:

1. What are potential safety concerns with the website that you could identify?
2. Did you come across any triggering content that could be conducive to harmful drug use?
3. How could this information lead to misunderstandings about MOUD?
4. How could this resource be used in lieu of seeing a healthcare provider?
5. What resources or information could lead misrepresent OUD and/or MOUD to friends and family of PLOUD ?
6. What are your thoughts on the “check-in” questions based on motivational interviewing? In what way could they be harmful or triggering?
7. How could the use of the stages of change model to structure the information in this website confuse people or deter them from engaging in MOUD treatment?
8. Are there any examples of stigmatizing, disrespectful or harmful language that are used on the website? If so, what safety-language could be used instead?

Final Question:

1. If you were to build a website for people with OUD, what is your idea or vision of it?
2. What would you add, what resources would you have?
3. How would you design it?

Thank you so much for your time and participation!
