# Supplementary material for: Assessing the Safety, User Acceptability, Dissemination, and Reach of a Comprehensive Web-Based Resource on Medications for Opioid Use Disorder (MOUD Hub): Protocol for a Development and Usability Study
Source: JMIR Res Protoc. 2024 Nov 7;13:e57065. doi: 10.2196/57065 (PMC11582488; doi:10.2196/57065)
Supplement: Multimedia Appendix 1 [file resprot_v13i1e57065_app1.docx]

**Multimedia Appendix 1**

**Section 1:**

Stages of Change

The *MOUD Hub* will welcome the users with a description of the website. From there, participants can choose which stage they are at and click on the respective wheel section. They will be taken to the stage-specific page containing resources that are useful for that stage. At the end of each stage, users will be met with a “check-in” that utilizes MI skills of open-ended questions to help users think about where they are at. The pre-contemplation and contemplation stages contain the most questions that relate to eliciting change talk, while in the latter stages contain questions that continue to motivate the change behavior and check-in on any areas that need attention. We added in a termination phase as our preliminary research found that people on MOUD often do not want to be on it for long periods of time and ask about tapering off it. While evidence indicates that treatment with MOUD is most effective when it is long-term, it is important to acknowledge individuals’ personal treatment objectives and to prevent harm in the process. Each stage also has friend and family specific resources and support. Some of these resources are duplicated across stages.

**Section 2:**

High-Fidelity snapshots of Webpages

Figure S1. Pre-contemplation Stage:

For the Pre-Contemplation Stage, the tab themes are Opioid Use Disorder, Harm Reduction, Overdose, HIV/Hepatitis C-Testing, and Check-In.

Figure S2. Contemplation Stage:

For the Contemplation Stage, the tab themes are My Options, Methadone, Buprenorphine, Naltrexone, What is Right for Me?, Friends and Family, and Check-In.

Figure S3. Preparation Stage:

For the Preparation Stage, the tab themes are Where to get MOUD, Recovery Support Groups, Transportation, Pharmacy, Cost of MOUD, Storing Medication, Treatment During Pregnancy, Preparing for Stigma, Who Can I call, Friends and Family, and Check-In.

Figure S4. Action Stage:

For the Action Stage, the tab themes are Planning Your Schedule, Coping Skills, Common Side Effects, Friends and Family, and Check-In.

Figure S5. Maintenance Stage:

For the Maintenance Stage, the tab themes are Apps for Support, MOUD Support Groups, Mental Health, Finding Activities, Friends and Family, and Check-In.

Figure S6. Recurrence Stage:

For the Recurrence Stage, the tab themes are Relapse-Treatment Attempts, How to Get Back on Treatment, Relapse Prevention plan, Friends and Family, and Check-In.

Figure S7. Termination Stage:

For the Termination stage, there are no tabs and the stage focuses more on what termination can look like and resources.

**Section 3:**

Disclaimers:

The website will also include disclaimers as we are not medical professionals. These include statements such as “The information provided on this site is for educational purposes only and does not substitute for professional medical advice. Please consult with a medical professional or healthcare provider if you are seeking medical advice, diagnoses, or treatment.”

We also include a disclaimer on language. “We have done our best to not use stigmatizing language in this website. Words that are presented in quotations are words that we do not agree with, but that have been present in the substance use field. Additionally, when we use the term recovery, we mean it is as a way to improve your quality of life.”

Lastly, we added in a disclaimer stating “We have collected information from other websites and compiled them here for you to look at.” This way people who use the website will know that the information is compiled from other resources that can be used.
