# Supplementary material for: Assessing the Safety, User Acceptability, Dissemination, and Reach of a Comprehensive Web-Based Resource on Medications for Opioid Use Disorder (MOUD Hub): Protocol for a Development and Usability Study
Source: JMIR Res Protoc. 2024 Nov 7;13:e57065. doi: 10.2196/57065 (PMC11582488; doi:10.2196/57065)
Supplement: Multimedia Appendix 2 [file resprot_v13i1e57065_app2.docx]

**Multimedia Appendix 2**

**Section 1:**

Snapshots of High-Fidelity Prototypes of Webpages

Figure S1. Pre-contemplation Stage:

For the Pre-Contemplation Stage, the tab themes are Opioid Use Disorder, Harm Reduction, Overdose, HIV/Hepatitis C-Testing, and Check-In.

Figure S2. Contemplation Stage:

For the Contemplation Stage, the tab themes are My Options, Methadone, Buprenorphine, Naltrexone, What is Right for Me?, Friends and Family, and Check-In.

Figure S3. Preparation Stage:

For the Preparation Stage, the tab themes are Where to get MOUD, Recovery Support Groups, Transportation, Pharmacy, Cost of MOUD, Storing Medication, Treatment During Pregnancy, Preparing for Stigma, Who Can I call, Friends and Family, and Check-In.

Figure S4. Action Stage:

For the Action Stage, the tab themes are Planning Your Schedule, Coping Skills, Common Side Effects, Friends and Family, and Check-In.

Figure S5. Maintenance Stage:

For the Maintenance Stage, the tab themes are Apps for Support, MOUD Support Groups, Mental Health, Finding Activities, Friends and Family, and Check-In.

Figure S6. Recurrence Stage:

For the Recurrence Stage, the tab themes are Relapse-Treatment Attempts, How to Get Back on Treatment, Relapse Prevention plan, Friends and Family, and Check-In.

Figure S7. Termination Stage:

For the Termination stage, there are no tabs and the stage focuses more on what termination can look like and resources.

**Section 2:**

Disclaimers:

The website will also include disclaimers as we are not medical professionals. These include statements such as “The information provided on this site is for educational purposes only and does not substitute for professional medical advice. Please consult with a medical professional or healthcare provider if you are seeking medical advice, diagnoses, or treatment.”

We also include a disclaimer on language. “We have done our best to not use stigmatizing language in this website. Words that are presented in quotations are words that we do not agree with, but that have been present in the substance use field. Additionally, when we use the term recovery, we mean it is as a way to improve your quality of life.”

Lastly, we added in a disclaimer stating “We have collected information from other websites and compiled them here for you to look at.” This way people who use the website will know that the information is compiled from other resources that can be used.
